# Diagnostic Potential of Exosomal HypoxamiRs in the Context of Hypoxia–Sumoylation–HypoxamiRs in Early Onset Preeclampsia at the Preclinical Stage

**DOI:** 10.3390/life12010101

**Published:** 2022-01-11

**Authors:** Vladislava Gusar, Angelika Timofeeva, Vitaliy Chagovets, Nataliya Kan, Mikhail Vysokikh, Maria Marey, Anna Karapetyan, Oleg Baev, Gennadiy Sukhikh

**Affiliations:** 1 Laboratory of Applied Transcriptomics, Federal State Budget Institution, “National Medical Research Center for Obstetrics, Gynecology and Perinatology Named after Academician V.I. Kulakov” of the Ministry of Healthcare of the Russian Federation, Oparin Str. 4, 117997 Moscow, Russia; v_timofeeva@oparina4.ru; 2 Laboratory of Proteomics and Metabolomics of Human Reproduction, Federal State Budget Institution, “National Medical Research Center for Obstetrics, Gynecology and Perinatology Named after Academician V.I. Kulakov” of the Ministry of Healthcare of the Russian Federation, Oparin Str. 4, 117997 Moscow, Russia; v_chagovets@oparina4.ru; 3 Directorat, Federal State Budget Institution, “National Medical Research Center for Obstetrics, Gynecology and Perinatology Named after Academician V.I. Kulakov” of the Ministry of Healthcare of the Russian Federation, Oparin Str. 4, 117997 Moscow, Russia; n_kan@oparina4.ru (N.K.); g_sukhikh@oparina4.ru (G.S.); 4 Laboratory of Mitochondrial Medicine, Federal State Budget Institution, “National Medical Research Center for Obstetrics, Gynecology and Perinatology Named after Academician V.I. Kulakov” of the Ministry of Healthcare of the Russian Federation, Oparin Str. 4, 117997 Moscow, Russia; m_vysokikh@oparina4.ru (M.V.); m_marey@oparina4.ru (M.M.); 5 Maternity Department, Federal State Budget Institution, “National Medical Research Center for Obstetrics, Gynecology and Perinatology Named after Academician V.I. Kulakov” of the Ministry of Healthcare of the Russian Federation, Oparin Str. 4, 117997 Moscow, Russia; a_karapetyan@oparina4.ru (A.K.); o_baev@oparina4.ru (O.B.)

**Keywords:** microRNA/miRNA, preeclampsia, hypoxamiRs, exosomes, sumoylation, SUMO, diagnostics

## Abstract

As the search for non-invasive preclinical markers of preeclampsia (PE) expands, the number of studies on the diagnostic potential of exosomes is growing. Changes in the partial pressure of oxygen caused by impaired uteroplacental perfusion in PE are a powerful inducer of increased production and release of exosomes from cells, which also determine their cargo. At the same time, the expression pattern of oxygen-dependent microRNAs (miRNAs), called “hypoxamiRs”, is modulated, and their packing into exosomes is strictly regulated by sumoylation. In connection therewith, we emphasize the evaluation of exosomal hypoxamiR expression (miR-27b-3p, miR-92b-3p, miR-181a-5p, and miR-186-5p) using quantitative RT-PCR, as well as SUMO 1–4 and UBC9 (by Western blotting), in pregnant women with early-onset PE. The findings show that miR-27b-3p and miR-92b-3p expression was significantly changed at 11–14 and 24–26 weeks of gestation in the blood plasma of pregnant women with early-onset PE, which subsequently manifested. High sensitivity and specificity (AUC = 1) were demonstrated for these miRNAs in the first trimester, and significant correlations with a decrease in hemoglobin (r = 0.71, *p* = 0.002; r = −0.71, *p* = 0.002) were established. In mid-pregnancy, the miR-27b-3p expression was found to correlate with an increase in platelets (r = −0.95, *p* = 0.003), and miR-92b-3p was associated with a decrease in the prothrombin index (r = 0.95, *p* = 0.003). Specific exomotifs of studied miRNAs were also identified, to which the sumoylated ribonucleoprotein hnRNPA2/B1 binds, carrying out their packaging into exosomes. The expression of conjugated SUMO 1 (*p* = 0.05), SUMO 2/3/4 (*p* = 0.03), and UBC9 (*p* = 0.1) was increased in exosomes at early-onset PE, and the expression of free SUMO 1 (*p* = 0.03) and SUMO 2/3/4 (*p* = 0.01) was significantly increased in the placenta, as an adaptive response to hypoxia. Moreover, SUMO 2/3/4 was negatively correlated with miR-27b-3p expression in the placenta. In conclusion, the diagnostic potential of exosomal hypoxamiRs mediated by sumoylation may form the basis for the development of combined specific targets for the treatment of early-onset PE, as hnRNPA2/B1 is a target of miR-27b-3p, and its sumoylation creates miR-27b-3p–hnRNPA2/B1–SUMO 1–4 cross-talk.

## 1. Introduction

In the context of great obstetric syndromes associated with the impaired transformation of uteroplacental vessels, preeclampsia (PE) continues to attract the close attention of researchers [1,2]. This is because PE is a syndrome causing multisystem damage, including the cardiovascular system, kidneys, liver, brain, and hemostasis system, and is the leading cause of maternal and perinatal morbidity/mortality, affecting 2 to 8% of pregnancies [3,4]. The triggering of a sequential cascade of processes underlying multifactorial pathogenesis (impaired trophoblast invasion, decreased uteroplacental blood flow and irregular placental perfusion, hypoxia/reoxygenation, oxidative stress of the placenta, the release of proinflammatory and antiangiogenic factors, activation of the coagulation system, endothelial dysfunction, and subsequent generalized inflammatory response) occurs early in pregnancy [4,5,6,7,8,9,10]. However, the clinical manifestation of PE is observed after 20 weeks, which complicates its early diagnosis and prognosis [11]. Despite the widely used screening tests for predicting the risk of developing PE in the first trimester of pregnancy, representing a combination of biochemical markers with biophysical monitoring of the fetus [11,12,13,14,15,16], the search for non-invasive diagnostic predictors oriented to various links of pathogenesis and opening up new possibilities in predicting PE continues.

Progress in understanding the molecular processes associated with both the regulation of the normal development of the placenta and its dysfunction have made it possible to focus scientists on the study of the predictive role of microRNAs (miRNAs), which are small non-coding molecules that are epigenetic modulators of a significant number of biological processes [17]. Of particular importance, these can circulate in extracellular fluids as part of microvesicles, apoptotic bodies, and exosomes, acting as mediators of intercellular interactions and therapeutic targets in placenta-associated diseases [18,19]. A number of studies have evaluated the diagnostic potential of placental miRNAs in the first trimester of pregnancy, both in the whole blood plasma of pregnant women [20,21,22,23] and circulating in exosomes [24,25,26,27,28,29]. The latter are small membrane-bound vesicles 40–100 nm in diameter, which are formed from the endosomal compartment, are released into the extracellular space by most cells, and contain various signaling molecules [18]. Notably, increased production and release of exosomes from cells is induced by oxygen deprivation, and associated oxidative stress and decreased pH [30]. Hypoxia essentially alters the molecular cargo of exosomes, determines the specific pattern of miRNAs called “hypoxamiRs”, and regulates their release after delivery and intracellular activity in the recipient cells [30,31,32,33]. It is important to note that the loading of miRNA into exosomes is strictly selective and is under the control of sumoylation, which is also one of the key mechanisms of cell adaptation in response to hypoxia and oxidative stress [34,35]. In particular, the sumoylation of HIF1 enhances its transcriptional activity [36].

Despite the active study of the prospects for use of exosomes as diagnostic markers in placental-associated diseases [25,28,37,38], data on the research of exosomal hypoxamiRs in PE are sporadic. [39]. In addition, there are no data on the exosomal content of proteins that carry out sumoylation and, accordingly, control the loading of miRNA into exosomes, although an increase in their expression in the placenta has been shown as well as an established link between global sumoylation and severe PE. [40]. Previously, we reported a significant increase in the expression of oxygen-sensitive miR-27b-3p, miR-92b-3p, miR-181a-5p, and miR-186-5p, selected according to deep sequencing data, in the blood plasma of pregnant women with early-onset PE at the time of delivery and their involvement in the regulation of signaling pathways mediated by hypoxia [41]. In the context of the available data, we found it interesting not only to evaluate the diagnostic potential of these hypoxamiRs in exosomes of pregnant women at early gestation (11–14 and 24–26 weeks) using logistic regression models but also to establish their predictive value associated with changes in the hemostatic system. In addition, the results of a pilot study in plasma exosomes of pregnant women with early-onset PE demonstrated an increased expression of the SUMO 1–4 and UBC9 proteins, probably in conjugation with the hnRNPA2/B1 protein, which loads miRNA into exosomes under the control of sumoylation. The obtained data made it possible to define the concept of “hypoxia–sumoylation–hypoxamiRs”, within which microRNAs induced by hypoxia and mediated by sumoylation can be considered as promising specific targets for therapeutic use in early-onset PE. The study of SUMO 1–4 proteins in exosomes of pregnant women with PE is a pilot study. To the best of the authors’ knowledge, this area has thus far been understudied.

## 2. Materials and Methods

### 2.1. Study Design and Patient Cohort

**Figure 1:**
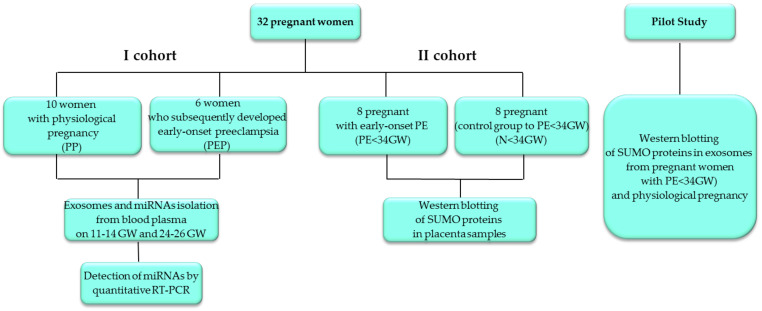
Flowchart of the study population.

This study included pregnant women who were under observation at the National Medical Research Center for Obstetrics, Gynecology, and Perinatology named after Academician V.I. Kulakov of the Ministry of Healthcare of the Russian Federation. The total sample of patients of reproductive age consisted of 32 pregnant women, divided into 2 cohorts (Figure 1). The first cohort (cohort I) included 16 pregnant women. Plasma samples of peripheral blood were obtained from them at 11–14 and 24–26 weeks of pregnancy to evaluate the expression of exosomal miRNA. Early-onset PE, with a severe course in the third trimester of pregnancy, manifested in 6 of them. The second cohort (cohort II) included pregnant women with early-onset PE (8 women) and pregnant women of the control group (8 women) of the corresponding gestational age to evaluate the expression of SUMO proteins in placenta samples. This cohort of pregnant women was used in our previous study to evaluate the expression of oxygen-sensitive miRNA [41]. The clinical characteristics of pregnant women included in the study are presented in Table 1, Table 2 and Table 3. To evaluate the expression of SUMO proteins in exosomes, a pilot study was carried out in groups of pregnant women (7 with early-onset PE and 7 with physiological pregnancy).

The estimation of fetal weight centiles is given in accordance with INTERGROWTH-21st (https://intergrowth21.tghn.org/translated-resources/) (accessed on 9 January 2022).

### 2.2. Exosome Purification and Isolation and miRNA Extraction from the Blood Plasma of Pregnant Women

Samples of blood plasma obtained during 11–14 and 24–26 weeks of pregnancy were collected into VACUETTE® tubes containing EDTA (BectonDickinson, Mississauga, ON, Canada). The samples were prepared according to the following protocol: whole blood was centrifuged at 300× *g*, 4 °C for 20 min, and then the supernatant was centrifuged at 16,000× *g* for 10 min. A total of 200 μL of the prepared plasma was used for isolation of exosomes, and 5.6 × 108 copies of cel-miR-39 (miScript Primer Assay, Qiagen, Hilden, Germany) were added to a plasma sample as an endogenous control for the efficiency of isolation and subsequent cDNA synthesis for quantitative RT-PCR. MiRNA was isolated using the exoRNeasy Midi Kit (cat. no: 77144, Qiagen). The extraction stages were carried out at an automatic station (QIAcube) in accordance with the protocols of the manufacturer Qiagen.

For Western blotting, exosomes were purified from blood samples and collected in VACUETTE® tubes containing EDTA (Becton Dickinson Mississauga, ON, Canada) according to the following protocol: whole blood was centrifuged at 2000× *g*, 4 °C for 15 min, and then the supernatant (plasma) was centrifuged at 100,000× *g*, 4 °C for 1 h by an ultracentrifuge (Optima XPN-100, Beckman Coulter Life Sciences, Indianapolis, IN, USA). The resulting precipitate was resuspended in 1× PBS (10× Phosphate Buffered Saline, cat. #1610780 Bio-Rad, Hercules, CA, USA) and centrifuged again under the same conditions. After repeated centrifugation, the pellet containing exosomes was resuspended in 50 μL of PBS and frozen for subsequent storage at −80 °C. Protein concentration was measured using spectrophotometer DS-11 (DeNovix Inc., Wilmington, DE, USA).

### 2.3. Real-Time Quantitative RT-PCR

The reverse transcription reaction was performed using the miScript II RT Kit (Qiagen). Quantitative PCR with the miScript SYBR Green PCR Kit (Qiagen) was performed using a StepOnePlus device (Applied Biosystems, Foster City, CA, USA) to determine the level of miRNA expression in the exosomes of blood plasma. The following RNA-specific sense primers were used: hsa-miR-27b-3p MIMAT0000419 (5′-TTCACAGTGGCTAAGTTCTGC, Tm = 52 °C), hsa-miR-92b-3p MIMAT0003218 (5′-TATTGCACTCGTCCCGGCCTCC, Tm = 52 °C), hsa-miR-181a-5p MIMA T0000256 (5′-AACATTCAACGCTGTCGGTGAGT, Tm = 56 °C) hsa-miR-186-5p MIMAT0000456 (5′-CAAAGAATTCTCCTTTTGGGCT, Tm = 52 °C), and cel-miR-39 (Tm = 55 °C). The stages were carried out in accordance with the Qiagen protocols. The threshold level of expression was Ct ≤ 37. The level of miRNA expression was determined by the 2−ΔΔCT method [42], using cel-miR-39 miScript Primer Assay (Qiagen) as an internal control for variations during the isolation of RNA, cDNA synthesis, and real-time PCR, and as a reference RNA.

### 2.4. Western Blotting

To determine the SUMO 1–4 proteins, samples of placental tissue (a cross-section through the maternal and fetal part of the placenta no more than 5 mm in thickness, obtained immediately after delivery) from pregnant women (cohort II) were used. Powdered tissue samples preliminarily ground in liquid nitrogen were homogenized in a RIPA Lysis Buffer System (sc-24948; Santa Cruz Biotechnology, Inc., Dallas, TX, USA). As a pilot study, the SUMO 1-4 and UBC9 in exosomes isolated from the blood plasma of 7 pregnant women with early-onset PE and 7 pregnant women with physiological pregnancy was also determined. Separation of proteins (20 μg per gel lane) was performed in Tris/Tricine/SDS Buffer (12.5%). The molecular weight marker was PageRuler™ Prestained Protein Ladder, 10 to 180 kDa (cat. #. 26617, Thermo Fisher Scientific, Waltham, MA, USA). Protein transfer to nitrocellulose membrane (0.45 µm, cat. no: 1620115 Bio-Rad, USA) was performed using Trans-Blot SD™ (cat. #. 170-3957, Bio-Rad, USA) in 10 mM CAPS + 10%C2H5OH, pH = 11. The membranes were blocked with 5% NFDM/TBST for 2 h. Incubation with primary antibodies: SUMO 1 (1:1000; ab32058, Abcam, Cambridge, UK), SUMO 2/3/4 (1: 100; sc-393144, Santa Cruz Biotechnology, USA), UBC9 (1:100; sc-271057, Santa Cruz Biotechnology, USA) and Actin (1:100; sc-376421; Santa Cruz Biotechnology, USA) were performed overnight (+4 °C). Secondary HRP-conjugated antibodies (goat anti-rabbit IgG-HRP: ab97051, Abcam, UK; goat anti-mouse IgG-HRP: sc-2031, Santa Cruz Biotechnology, USA) were incubated for 1 h (RT). A SuperSignal West Femto Maximum Sensitivity Substrate Kit (cat. no: 34096, Thermo Scientific™, USA) was used as a detection reagent. Densitometric analysis was performed using Bio-Rad ImageLab 6.0 software. The expression of tissue proteins SUMO 1–4 was normalized to that of actin, and the level of SUMO 1–4 and UBC9 in exosomes was normalized to the concentration of total protein.

### 2.5. Statistical Analysis

The statistical significance of the difference between the clinical parameters and the levels of miRNA expression in the groups under study was assessed by the Wilcox–Mann–Whitney test using scripts written in the R language (https://www.R-project.org/) (accessed on 9 January 2022). Logistic regression models for miRNA expression were created to test the possibility of using them as biomarkers. The efficiency of the created models was evaluated by ROC analysis. The normality of clinical parameters distribution was evaluated by the Shapiro–Wilk test. Statistical analysis was performed using Student’s test with a normal distribution of the parameter and using the Mann–Whitney test when the distribution did not correspond to the law of normal distribution. To describe quantitative data having a normal distribution, the mean value (M) and standard deviation (SD) in the M ± SD format were used. In the case of non-normal distribution, the median (Me) and quartiles Q1, Q3 in the format Me (Q1–Q3) were used. The Spearman nonparametric rank correlation method was used to evaluate the relationship between the expression level of studied mRNAs, clinical parameters, and protein level.

## 3. Results

### 3.1. Evaluation of Oxygen-Sensitive miRNA Expression in Exosomes at 11–14 and 24–26 Weeks of Gestation

**Figure 2:**
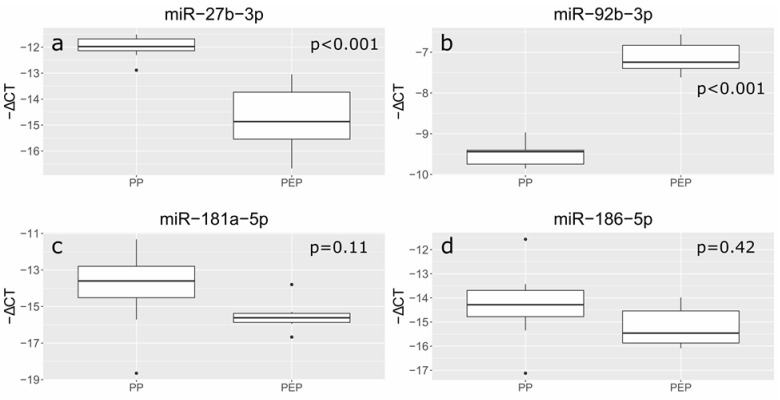
Comparative analysis of the expression levels for miR-27b-3p (**a**), miR-92b-3p (**b**), miR-181a-5p (**c**), and miR-186-5p (**d**) in the exosomes of blood plasma from pregnant women at 11–14 GW. The box diagram shows the medians of −ΔCt values (relative quantification data), the first and third quartiles, the edges of the statistically significant sample, and the dots denote the emissions. PP is a physiological pregnancy. PEP is a pregnancy with preeclampsia-onset.

MiR-27b-3p, miR-92b-3p, miR-181a-5p, and miR-186-5p were selected for the experiment as we previously determined the altered expression in the peripheral blood plasma of pregnant women with early-onset PE during delivery. The expression of the above miRNAs was evaluated in exosomes of 16 pregnant women (cohort I) at gestational periods 11–14 (GW, gestational weeks) and 24–26 (GW, gestational weeks) [41]. Six of the sixteen pregnant women included in the study subsequently developed severe early-onset PE. In this connection, we compared the miRNA expression at the corresponding stages of gestation in the PEP group with the PP group. The expression of miR-27b-3p was significantly reduced (fold change = 0.20; *p* < 0.001), and the expression of miR-92b-3p was increased (fold change = 7.0; *p* < 0.001) in the PEP group relative to the PP group at 11–14 weeks gestation. There were no significant differences in the expression levels of miR-181a-5p (*p* = 0.11) and miR-186-5p (*p* = 0.42) currently (Figure 2).

**Figure 3:**
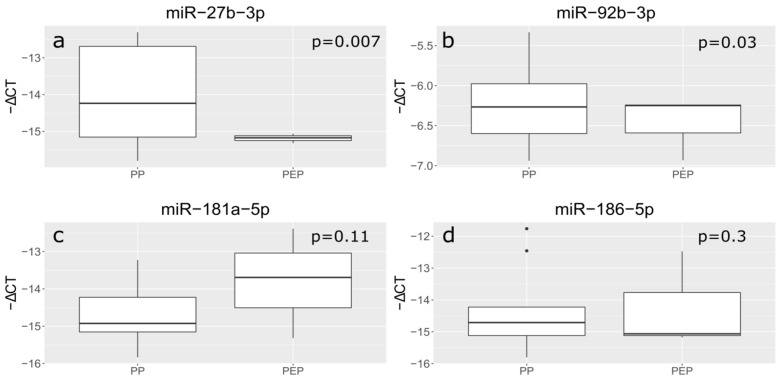
Comparative analysis of the expression levels for miR-27b-3p (**a**), miR-92b-3p (**b**), miR-181a-5p (**c**), and miR-186-5p (**d**) in the exosomes of blood plasma from pregnant women at 24–26 GW. The box diagram shows the medians of −ΔCt values (relative quantification data), the first and third quartiles, the edges of the statistically significant sample, and the dots denote the emissions. PP is a physiological pregnancy. PEP is a pregnancy with preeclampsia onset.

The miR-27b-3p expression (fold change = 0.26; *p* = 0.007), as well as miR-92b-3p expression (fold change = 0.51; *p* = 0.03), was significantly reduced in the PEP group compared to the PP group at 24–26 weeks of gestation (Figure 3).

### 3.2. ROC Analysis of Oxygen-Sensitive Exosomal miRNAs at 11–14 and 24–26 Weeks of Gestation and Their Correlation with Clinical Data of Pregnant Women

To assess the possibility of using oxygen-dependent miR-27b-3p and miR-92b-3p as potential diagnostic markers for the development of early-onset PE at the corresponding gestational age, logistic regression models were created. It should be noted that when assessing the predictive significance of miR-27b-3p and miR-92b-3p at 11–14 GW, we obtained the highest AUC values, both for each miRNA individually and in their combination (Table 4).

Considering the results, we searched for correlations between the expression of the studied miRNAs in exosomes and clinical parameters in groups of pregnant women using the Spearman non-parametric rank correlation method. Correlations were assessed, in general, by groups, and separately in the PEP group (Table 5). Clinical parameters included: blood pressure, general blood test, hemostatic profile, combined first-trimester screening data in PE (11–14 GW), and Doppler data (24–26 GW) (Table 5).

The analysis revealed a positive correlation between miR-27b-3p expression and the hemoglobin level of pregnant women at 11–14 GW (r = 0.71, *p* = 0.002) and 24–26 GW (r = 0.66, *p* = 0.05). At the same time, the hemoglobin level negatively correlated with miR-92b-3p expression at 11–14 GW (r = −0.71, *p* = 0.002). It should be noted that within the PEP group, a negative correlation was found between miR-27b-3p expression and the platelet at the 24–26 GW (r = −0.95, *p* = 0.003), and the miR-92b-3p expression was positively correlated with the prothrombin index (r = 0.95, *p* = 0.003).

### 3.3. MiRNA Exomotif Analysis

Based on the data on the presence of specific exomotifs in miRNA, which are necessary for sorting the latter into exosomes by way of binding their exomotifs to the hnRNPA2/B1 packaging protein [34], we analyzed the sequences of the studied hypoxamiRs for the presence of these exomotifs. A variation of GGCC exomotif (16–19 nt) in the 3 ‘sequence of mature miR-92b-3p and a variation of TGGG exomotif (17–20 nt) in the 3’ sequence of miR-186-5p were identified. Interestingly, when analyzing the miR-27b-3p sequence, we did not reveal any of the exomotif variations proposed by the authors. However, in its 5’-sequence (9–12 nt), a GGCT variation was found, differing by one nucleotide from the exomotif GGCC of miR-92b-3p. In the sequence of mature miR-181a-5p, one of the motifs, AACAT (1–5 nt), characteristic of miRNAs differentially expressed in cells, but not in exosomes, was identified [34].

### 3.4. SUMO Expression in Exosomes and Placenta Tissue—Evaluation of Correlations with HypoxamiR Expression in Early Onset PE

**Figure 4:**
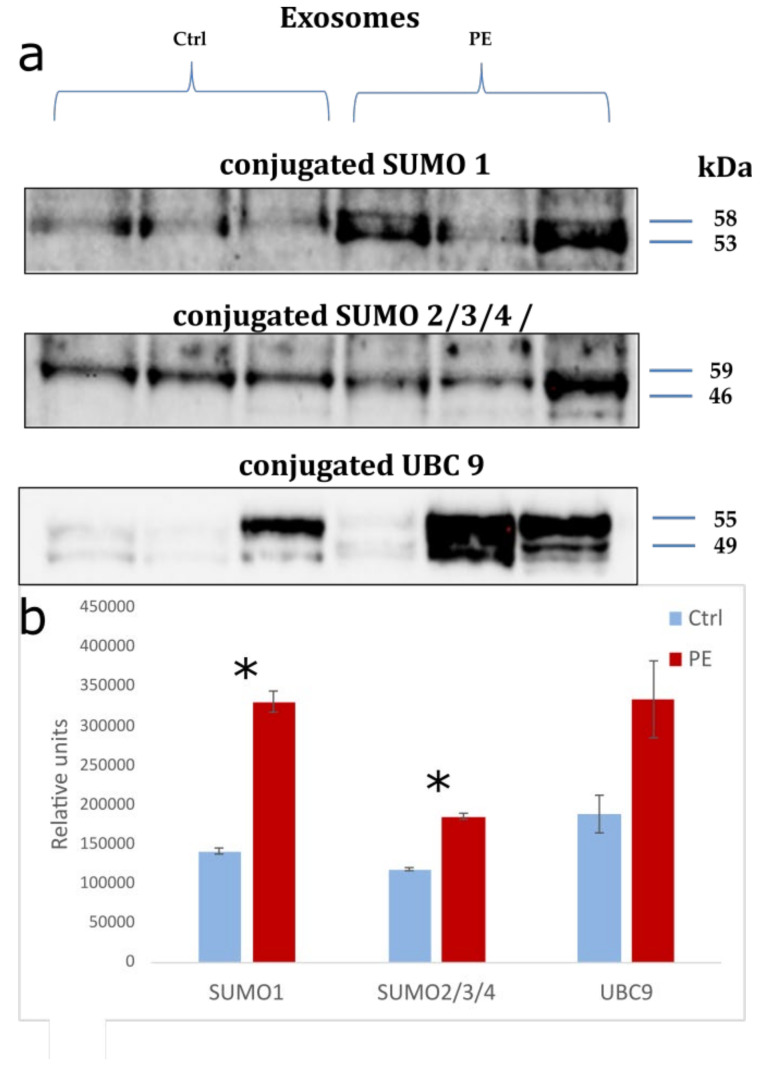
(**a**) Western blot analysis of SUMO 1-4 and UBC9 in exosomes in pregnant women with early-onset PE (<34 GW) and control samples (Ctrl); (**b**) Total densitometry of conjugated proteins was quantified and normalized to total protein concentration. Data are presented as the mean ± SE of 7 samples in each group. The symbol (*) denotes the *p*-value < 0.05.

The expression of SUMO 1–4 and UBC9 in exosomes isolated from the blood plasma of pregnant women with early-onset PE (7 women) and control samples (7 women) was estimated using Western blotting as a pilot study. SUMO 1–4 and UBC9 were found in conjugated forms (Figure 4a). Meanwhile, the expression of conjugated SUMO 1 (*p* = 0.05) and SUMO 2/3/4 (*p* = 0.03) in pregnant women with early-onset PE was significantly increased relative to the control. The expression of conjugated UBC9 (~55 and ~49 kDa) showed an upward trend, but without a statistically significant difference (*p* = 0.1) (Figure 4b). Interestingly, in exosomes of pregnant women with PE, conjugated SUMO 1 and SUMO 2/3/4 were expressed in the form of two fragments differing in molecular weight (~53 and ~58 kDa; ~46 and ~59 kDa, respectively), while in the control group, only one fragment was expressed (~56 kDa; ~48 kDa, respectively).

**Figure 5:**
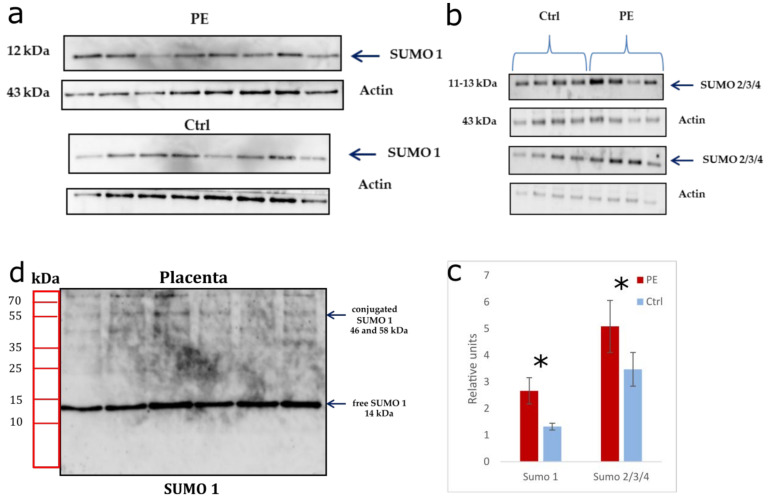
Western blot analysis of SUMO 1 (**a**) and SUMO 2/3/4 (**b**) in placental tissue in early-onset PE (<34 GW) and age-matched controls Ctrl (<34 GW). Total densitometry of SUMO 1–4 were quantified and normalized to loading control actin. Data are presented as the mean ± SE of 8 placentas in each group; (**c**) Western blot analysis of membrane with SUMO 1 in free and conjugated forms in placental tissue in early-onset PE (**d**). The symbol (*) denotes the *p*-value < 0.05. Interestingly, the miR-27b-3p and miR-186-5p expression were significantly reduced (*p* < 0.003) in the placenta from pregnant women with early-onset PE (cohort II) as compared to the control group of the corresponding gestational age in our previous study [42]. In this regard, using the nonparametric Spearman rank correlation method, a significant correlation was established between the change in SUMO 2/3/4 expression and oxygen-sensitive miR-27b-3p, miR-186-5p in pregnant women with early-onset PE (Table 6).

Considering that the sumoylation is involved in the formation of an adequate cellular response to hypoxia and the triggering of the signaling cascade of reactions, we estimated the expression of the SUMO 1 and SUMO 2/3/4 in the placenta in pregnant women with early-onset PE (cohort II) (Figure 5a,b). An increase in SUMO 1 (2.66 ± 0.49, *p* = 0.03) and SUMO 2/3/4 (5.08 ± 0.97, *p* = 0.01) was observed in PE compared with the control group of the corresponding gestational age (1.32 ± 0.12, *p* = 0.03; 3.47 ± 0.63, *p* = 0.01) (Figure 5c). It should be noted that SUMO 1 was present in the placenta, in both free (~14 kDa) and two conjugated forms (~46 kDa and ~58 kDa) (Figure 5d).

The expression values of miR-27b-3p (r = −0.51, *p* = 0.04) and miR-186-5p (r = −0.52, *p* = 0.04) negatively correlated with the level of SUMO 2/3/4. Therefore, with an increase in the level of SUMO 2/3/4, the expression of the above miRNAs decreases. No significant correlations were found in SUMO 2/3/4 with miR-92b-3p and miR-181a-5p expression. We also did not find significant correlations in SUMO 1 with these miRNAs’ expression levels.

### 3.5. Signaling Pathway Analysis

**Figure 6:**
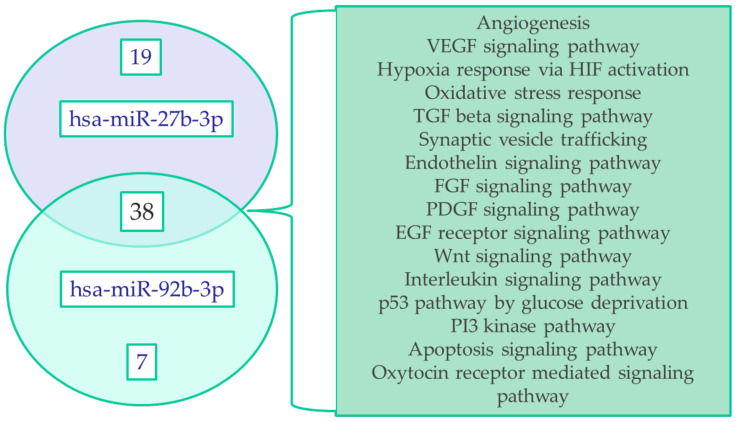
Venn Diagram. The diagram shows the common signaling pathways mediated by hypoxia (*p*-value ≤ 0.05), common for the studied miRNAs, in the potential regulation of which their target genes are involved. The numbers indicate the number of paths.

Taking into account the established correlations between miR-27b-3p and miR-92b-3p expression and the clinical data of pregnant women, we found it interesting to evaluate the participation of these miRNAs in signaling pathways. Using bioinformatics databases (MiRTarBase4.5, DAVID6.8, PANTHER14.1), we conducted a relevant search (Figure 6).

## 4. Discussion

It has been experimentally established that in the early stages of pregnancy, fluctuations in oxygen tension cause the release of exosomes from cytotrophoblast cells, the functional activity of which can cause phenotypic changes in smooth muscle and endothelial cells of the spiral arteries of the uterus. Depending on the prevailing oxygen pressure, these fluctuations can promote or prevent vascular remodeling [43]. At the same time, pathologies associated with the placenta are also characterized by an increase in the concentration of exosomes in the mother’s bloodstream already in the early stages of gestation in comparison with physiologically proceeding pregnancy [25]. In a string of studies, the differential expression profiles of exosomal miRNAs in early pregnancy were identified as biomarkers in PE and fetal growth restriction. The researchers’ focus was mainly on the validation of placental-specific miRNAs [20,21,22,24,25,44]. It should be noted that initially, miRNAs regulated by hypoxia were studied in tumors of various origins, which made it possible to establish the tissue-specificity of their expression, as well as the dependence on the duration of exposure to oxygen deprivation [45]. Single studies, in particular, that conducted by Biróa et al., have demonstrated an increase in the total amount of exosomal miRNA and, as the most studied, hypoxamiR miR-210, which is highly expressed in the exosomes of women with severe PE [39]. Regarding the available data and based on the results of our previous studies, which demonstrated differences in the expression of oxygen-dependent miRNAs (miR-27b-3p, miR-92b-3p, miR-181a-5p, and miR-186-5p) in the blood plasma of pregnant women with early-onset PE at the time of delivery [41], we aimed to estimate the expression of the latter in exosomes at early gestation in the present study. In this regard, a significant decrease in miR-27b-3p expression was revealed at the 11–14 GW and 24–26 GW, while miR-92b-3p expression changed in different directions: it increased at the 11–14 GW and decreased at the 24–26 GW in pregnant women with early-onset PE. We hypothesized that the observed multidirectional changes in their expression may be associated with the regulation of various targets by these miRNAs involved in the process of trophoblast differentiation and stimulation of angiogenesis under conditions of low oxygen pressure, and then, in the formation of placental circulation, under conditions of increased oxygen concentration [5]. The hypoxic effects observed during abnormal placentation are mainly mediated by the transcription factor induced by the hypoxia, HIF1 [31]. HIF1 can exert multilevel effects on the hypoxamiRs network by directly binding to the HRE (hypoxia regulatory elements) located in the promoter regions of a number of miRNAs. However, a significant number of miRNAs under conditions of low oxygen availability can be regulated by HIF-independent pathways, including modulation of inflammatory responses and activation of endothelial cells. [31]. Using bioinformatics databases, we determined that the target genes of the studied hypoxamiRs are involved in the signaling cascade of the response to hypoxia, as well as signaling pathways mediated by hypoxia.

Under the influence of hypoxia, the cargo of exosomes also changes, causing differences in the miRNA profiles between donor and recipient cells [30,33]. It is important to emphasize that the packing of miRNA into exosomes is an active process. Recently, a mechanism for their selective sorting was discovered, carried out by heterogeneous ribonucleoproteins (hnRNPA2/B1 and hnRNPA1), and strictly regulated by one of the types of post-translational modification called sumoylation [34]. Moreover, sumoylation is necessary for adequate activation of the response to hypoxia and triggering subsequent adaptation processes involving certain proteins by their covalent attachment to SUMO (small ubiquitin-like modifier proteins) [35]. Sumoylation of hnRNPA2/B1 by SUMO 1 is a prerequisite for its recognition and binding to specific exomotifs of miRNA for their subsequent loading into exosomes whereas inhibition of sumoylation can disrupt the binding of the protein to miRNA [34]. Based on the above data, we evaluated the expression of SUMO 1 in exosomes of pregnant women with early-onset PE, for which a pilot study was carried out. The obtained results demonstrated a significant increase in the expression of conjugated SUMO 1. We supposed that SUMO 1 in exosomes is in a complex with hnRNPA2/B1 packing protein. This is consistent with the studies of Villarroya-Beltri et al., confirming a binding of hnRNPA2/B1 with the miRNA exomotifs and subsequently sorting into exosomes [34]. An even more interesting finding was the presence in exosomes of other isoforms SUMO 2/3/4, and UBC9 probably also conjugated to hnRNPA2/B1. The UBC9 is a unique enzyme involved in the multi-step sumoylation process. It promotes the formation of an isopeptide bond between SUMO and the target protein [46]. At the same time, the expression of conjugated SUMO 2/3/4 were also significantly increased in exosomes of pregnant women with early-onset PE relative to the control, and a tendency to an increase in conjugated UBC9 was observed without a statistically significant difference. It is interesting to note that the conjugated SUMO 1–4 included two fragments that differed in molecular weight in pregnant with PE, whereas only one of those fragments was presented in the control. We assumed this was associated with a certain activity of types A2 and B1 of packing protein hnRNP, which form complexes with the indicated proteins in norm and PE. It should be noted that these data were first obtained when studying exosomal content in pregnant women with PE.

Villarroya-Beltri et al. experimentally proved that a number of miRNAs in T cells are specifically sorted into exosomes, while other miRNAs are retained by the cell, regardless of their activation status. The effect of suppressing or increasing the expression of hnRNPA2/B1 has been shown, whereby it affects its specific binding only to those miRNAs that contain exomotifs [34]. Recent research by Devor et al. also determined that miRNAs expressed in exosomes in the first trimester in pregnant women with subsequently manifested PE are, as a rule, evolutionarily older, and their mature sequences differ from those that are not loaded into exosomes [29]. Based on the above data, we identified the corresponding exomotifs in hypoxamiRs, according to those found in the research carried out by Villarroya-Beltri C. et al. [34].

Sumoylation affects the stability, activity, and intracellular localization of transcription factors that regulate trophoblast differentiation, in particular, GCM1, DREAM, HIF-1α [47], and angiogenesis (VEGF), and forms an inflammatory response (NF-κB) [35]. A unique subcellular distribution of SUMO isoforms in the trophoblast layers was shown during physiological pregnancy, and under conditions of hypoxia and oxidative stress, an increase in the level of the latter in the placenta tissue [47]. However, data on the study of sumoylation in placental-related diseases are limited [40]. In particular, the studies of Baczyk et al. revealed a significant increase in the level of SUMO 1 and SUMO 2/3 in the placenta in severe early-onset PE [40]. Crucially, the proteins that mediate metabolic adaptation to hypoxia and are targets of HIF1α have increased expression and conjugation with SUMO [48]. Considering our results on the estimation of the SUMO 1–4 isoforms expression in exosomes of pregnant women with early-onset PE, it seemed interesting to evaluate the expression of these proteins in the placenta. A significant increase in the expression of SUMO 1 and SUMO 2/3/4 in the placenta tissue of pregnant women with early PE was revealed by Western blotting. It is consistent with the above data of other authors, demonstrating global sumoylation in response to oxygen deprivation in the placenta of pregnant women with early-onset PE. Beyond that, we found SUMO 1 expression in the placenta, both in free and conjugated forms.

It should be noted that cross-talk between sumoylation and hypoxamiRs in PE is one of the unexplored aspects. Interaction in the context of “miRNA–target–sumoylation” has been demonstrated earlier in a few studies not related to placental pathologies. In particular, miR-146a overexpression significantly reduces the SUMO 1 expression and conjugated with SERCA2a, plays a critical role in patients with heart failure [49]. Suppression of the miR-200 and/or miR-182 family expression in the SHSY5Y human neuroblastoma cell line and the primary culture of rat cortical neurons, increases global sumoylation and cell resistance to death under conditions of oxygen-glucose deprivation [50]. In connection therewith, this analysis established a significant correlation between an increased SUMO 2/3/4 with a decrease in miR-27b-3p and miR-186-5p expression in the placenta in early-onset PE. It is important to emphasize that SUMO 2 is regulated by miR-186-5p (https://www.genecards.org/) (accessed on 9 January 2022). Therefore, the revealed correlations represent an example of cross-talk between sumoylation and hypoxamiRs in early-onset PE, which may reflect pathological processes in the placenta mediated by hypoxia. Moreover, sumoylation of hnRNPA2/B1 creates cross-talk “miR-27b-3p–hnRNPA2/B1–SUMO 1”, since hnRNPA2/B1 is a target of miR-27b-3p (https://www.genecards.org/) (accessed on 9 January 2022) that allows us to consider this context as combined specific targets for further research and therapeutic use in early-onset PE. Likely, changes in the expression of the miR-27b-3p may also affect the binding of hnRNPA2/B1 with other miRNAs and their packing into exosomes. However, given that miR-27b-3p is not the only regulatory miRNA for hnRNPA2/B1, it can be assumed that the relationship “miRNA–target–sumoylation–miRNA packing into exosomes” is not linear.

MiRNAs exposed to various stress stimuli differ in their profile in exosomes, which may indicate their relationship with certain pathological conditions [51] and arouse undoubted practical interest. The predictive value of miRNA with an assessment of the severity and risk of developing clinical manifestations in placental-related diseases is confirmed by the works of a number of authors [20,21,22,24]. In our research, an assessment of the usefulness of hypoxamiRs miR-27b-3p and miR-92b-3p as potential diagnostic markers for the development of early-onset PE before its clinical manifestation demonstrated high sensitivity and specificity at 11–14 weeks of gestation, as for each miRNA individually and in their combination (Table 4). Moreover, correlations were established between these miRNA expressions and clinical parameters in groups of pregnant women. In the early stages of gestation (11–14 GW), miR-27b-3p and miR-92b-3p expression were associated with a decrease in the hemoglobin concentration of pregnant women that may be caused by hypoxia. As reported by Penha-Silva et al., erythrocytes with a smaller volume and low hemoglobin level are more stable under hypoosmotic conditions, which is considered by the author as a compensatory decrease in cerebral blood flow in PE [52]. The miRNA expression was highly correlated with indices of the hemostasis system, in particular, miR-27b-3p, with an increase in the platelet, miR-92b-3p, with a decrease in the prothrombin index in pregnant women from the PEP group in mid-pregnancy (24–26 GW). It was shown previously that platelet activation occurs under the influence of hypoxia, while their phenotype, resistance to inhibition by antiplatelet agents, proteomic, and transcriptomic profiles change in patients with progressive peripheral artery disease [53,54]. In particular, studies by Miao et al. revealed suppression of miR-27b-3p expression in platelets after thrombin stimulation in vitro, which promotes de novo synthesis of TSP-1, exhibiting anti-angiogenic effects. At the same time, an increase in its expression inhibits the synthesis of TSP-1, enhancing the angiogenic activity of mature platelets [55]. According to Kaudewitz et al., miR-27b-3p expression correlates with platelet-activating factors (PF4 and PPBP) [56]. In a study by Hao et al., a decrease in miR-92b-3p expression under conditions of hypoxia suppresses the proliferation of pulmonary arterial smooth muscle cells in pulmonary arterial hypertension [57]. Moreover, a potential target of miR-92b-3p is PAFAH1B1 (https://www.genecards.org/) (accessed on 9 January 2022), a regulatory subunit of type I platelet-activating factor (PAF), which is involved in PAF inactivation. It is important to note that exosomal miRNAs, when transferred to recipient cells, can mediate functional effects by altering gene expression [58]. And in a series of papers, such effects have been studied, in particular, in the enhancement of angiogenesis [59] and endothelial dysfunction in PE [60]. In this regard, and considering the correlations indicated above, the differential expression of hypoxamiRs at 11–14 GW and 24–26 GW periods may be considered as a potential marker of disorders in the hemostasis of pregnant women with early-onset PE before its clinical manifestation.

## 5. Conclusions

**Figure 7:**
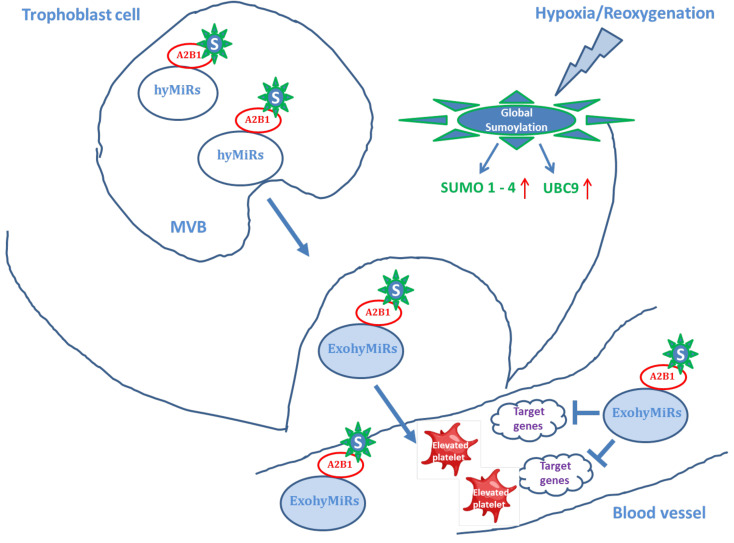
Schematic representation of the “hypoxia—sumoylation–hypoxamiRs” concept. HyMiRs are hypoxaMiRs. A2B1 is a heterogeneous ribonucleoprotein (hnRNPA2/B1). S is a SUMO 1 protein. MVB is a multivesicular body. ExohyMiRs are exosomal hypoxaMiRs.

In general, the results of our study can be summarized in the form of the concept—“hypoxia–sumoylation–hypoxamiRs”, the essence of which boils down to the following: inadequate remodeling of placental vessels in PE in early gestation induces fluctuations in the partial pressure of oxygen, resulting in global sumoylation, as an adaptive response by the body to hypoxia. Alongside, hypoxia contributes to a change in the expression and increased secretion of hypoxamiRs, the loading of which into exosomes is also mediated by sumoylation and is carried out by binding the sumoylated hnRNPA2/B1 protein to their specific exomotifs (Figure 7). At the same time, hypoxamiRs released by trophoblast cells into the mother’s blood flow, thereby changing the expression of target genes involved in the signaling cascade of the response to hypoxia, can mediate functional effects associated with changes in the hemostasis of pregnant women, prior to the clinical manifestation of PE.

## Figures and Tables

**Table 1:** The clinical characteristics of pregnant women (cohort I).

	Pregnant Women Cohort		
	PP (*n* = 10)	PEP (*n* = 6)	*p*-Value
Maternal age	28.5 ± 2.12	34.17 ± 6.59	0.09
BMI	21.07 ± 2.4	24.5 ± 3.58	0.07
History of PE, *n* (%)	0	3 (50)	-
Gestational age at the time of delivery, weeks	39.4 ± 1.09	34.9 ± 2.02	0.001
Spontaneous labor, *n* (%)	5 (50)	-	-
Cesarean section, *n* (%)	5 (50)	6 (100)	0.1
Birth weight, grams (centiles)	3419.9 ± 413.7	2071.5 ± 661.2	0.002
Apgar 1 score	7 (7–7)	8 (8–8)	<0.001
Apgar 5 score	9 (9–9)	8 (8–8)	<0.001

PP is a physiological pregnancy. PEP is a pregnancy with preeclampsia onset. BMI is a body mass index. For the normal distribution, the mean value (M) and standard deviation (SD) in the M ± SD format were used. In the case of non-normal distribution, the median (Me) and quartiles Q1, Q3 in the format Me (Q1–Q3) were used.

**Table 2:** The clinical characteristics of pregnant women cohort at 11–14 and 24–26 gestational weeks (cohort I).

KERRYPNX	11–14 GW		*p*- Value	24–26 GW		*p*- Value
	PP (*n* = 10)	PEP (*n* = 6)		PP (*n* = 10)	PEP (*n* = 6)	
Time of sampling	12.3 ± 0.67	12.3 ± 0.63	0.39	24.9 ± 0.73	24.5 ± 0.57	0.35
Systolic blood pressure (110–130 mmHg)	105 (100–110)	110 (110–110)	0.20	100 (92.5–110)	131 (115–138)	0.004
Diastolic blood pressure (65–80 mmHg)	60 (60–70)	70 (70–70)	0.07	60 (60–68.7)	82 (72.5–88.5)	0.003
Platelet level (150–400 * 10^9^ c/L)	228 (201–246)	256 (256–257)	0.09	254 (240–267)	226 (222–230)	0.01
PAPP-A (ME/л) b-HCG (ME/л)	3.2 ± 1.51 54.1 ± 26.2	1.89 ± 0.9 45.4 ± 31.4	0.04 0.58	NA NA	NA NA	- -
Ultrasonography:						
CRL	59.6 ± 3.7	57.5 ± 5.6	0.44	NA	NA	-
NTS	1.5 (1.4–1.6)	1.4 (1.3–1.4)	0.09	NA	NA	-
PI UtA	1.6 ± 0.3	1.9 ± 0.4	0.11	NA	NA	-
PI DV	1.03 ± 0.07	1 ± 0.06	0.4	NA	NA	-
Dopplerometry:						
PI UtA	NA	NA	-	0.92 ± 0.16	1.07 ± 0.25	0.24
PI UA PI MCA	NA NA	NA NA	- -	1.09 ± 0.15 2.45 (2.45–2.45)	1.35 ± 0.14 1.63 (1.63–1.63)	0.005 <0.001

GW is a gestational week. PP is a physiological pregnancy. PEP is a pregnancy with preeclampsia onset. PAPP-A is a pregnancy-associated protein A. b-HCG is a human chorionic gonadotropin; subunit b. CRL is a crown-to-rump length. NTS is a nuchal translucency scan. PI UtA is a Pulsatility Index of the uterine artery. PI UA is a Pulsatility Index of the umbilical artery. PI MCA is a Pulsatility Index of the middle cerebral artery. PI DV is a Pulsatility Index of ductus venosus. NA is not analyzed. For the normal distribution, the mean value (M) and standard deviation (SD) in the M ± SD format were used. In the case of non-normal distribution, the median (Me) and quartiles Q1, Q3 in the format Me (Q1–Q3) were used.

**Table 3:** The clinical characteristics of pregnant women (cohort II).

	Pregnant Women with PE < 34 GW (*n* = 8)	Control Group N < 34 GW (*n* = 8)	*p*-Value
Gestational age at the time of delivery, weeks	29 ± 3	29 ± 3	0.8
Manifestation PE, weeks	26 ± 4	absent	-
Systolic blood pressure (110–130 mmHg)	153.1 ± 16.2	115 ± 4.6	<0.001
Diastolic blood pressure (65–80 mmHg)	102.5 ± 12.2	72.5 ± 4.4	<0.001
Proteinuria (0–0.2 g/L)	2.6 ± 1.9	absent	-
Peripheral edema, *n* (%)	3 (37.5%)	absent	-
Ratio of placental dysfunction markers (sFLT-1/PLGF; 1.5–7)	386.3 ± 266.2	NA	-
Platelet level (150–400 * 10^9^ c/L)	153.5 ± 65.7	246.5 ± 62.9	0.01
Liver function test:			
ALT level (0–40 u/L)	50.3 ± 64.3	NA	-
AST level (0–40 u/L)	65.1 ± 75.2	NA	-
Birth weight, grams (centiles)	1345.7 ± 970.6	868.7 ± 533.8	0.2

NA here means “not analyzed”. GW denotes a gestational week. For the normal distribution, the mean value (M) and standard deviation (SD) in the M ± SD format were used. In the case of non-normal distribution, the median (Me) and quartiles Q1, Q3 in the format Me (Q1–Q3) were used.

**Table 4:** HypoxamiRs predictive values at 11–14 and 24–26 GW.

	miR-27b-3p + miR-92b-3p	
	11–14 GW	24–26 GW
AUC	1	0.83
ROC curve, *p*-value	<0.001	0.05
Sensitivity	1	1 (0.66–1)
Specificity	1	0.8 (0.5–1)
Cutoff	0.5	0.32
PPV	1	0.6 (0.37–1)
NPV	1	1
TP	6 (6–6)	3 (2–3)
TPR	1	1
FPR	0	0.3
TNR	1	0.7
FNR	0	0
ACC	1	0.76 (0.74–0.79)

PPV is a positive predictive value. NPV is a negative predictive value. TP is a true positive. TPR is a true positive rate. FPR is a false negative rate. TNR is a true negative rate. FNR is a false negative rate. ACC is the accuracy of the measurements. GW is a gestational week. The median (Me) and quartiles Q1, Q3 are in the format Me (Q1–Q3).

**Table 5:** The results of a correlation of miRNA expression in exosomes with clinical data of pregnant women.

		11–14 GW		24–26 GW	
miRNA (−ΔCt)	Parameter	r *	*p* **	r *	*p* **
miR-27b-3p	Hb level ‡	0.71	0.002	0.66	0.05
	Platelet Δ	-	-	−0.95	0.003
miR-92b-3p	Hb level ‡	−0.71	0.002	-	-
	Prothrombin index Δ	-	-	0.95	0.003

The ‡ symbol denotes the correlation parameters when comparing the PP vs. PEP groups, the Δ symbol denotes the correlation parameters obtained only in the PEP group. GW is a gestational week. * r is a Spearman rank correlation coefficient; ** *p* is the statistical significance of correlation (*p* < 0.05).

**Table 6:** The results of a correlation of SUMO 1–4 with miRNA expression level in placental tissue in early-onset PE.

	miR-27b-3p (−ΔCt)		miR-92b-3p (−ΔCt)		miR-181a-5p (−ΔCt)		miR-186-5p (−ΔCt)	
	r *	*p* **	r *	*p* **	r *	*p* **	r *	*p* **
**SUMO 1**	0.17	0.5	0.34	0.1	0.22	0.4	0.21	0.4
**SUMO 2/3/4**	−0.51	0.04	0.1	0.5	0.32	0.23	−0.52	0.04

* r is a Spearman rank correlation coefficient; ** *p* is the statistical significance of correlation (*p* < 0.05).

## Data Availability

The datasets used and/or analyzed in the current study are available from the corresponding author on reasonable request.
